# Microstructural alterations of the trigeminal ganglion in chronic ocular surface pain patients: A diffusion MRI study

**DOI:** 10.1016/j.neuroimage.2025.121309

**Published:** 2025-06-18

**Authors:** Alpen Ortug, Nicholas Reyes, Anat Galor, David Valdes-Arias, Ema Karakoleva, Cameron Talbert, Nicholas J. Pondelis, Pradip Pattany, Elizabeth Felix, Scott Holmes, David Zurakowski, Barry Sessle, Emi Takahashi, Eric A. Moulton

**Affiliations:** a Department of Radiology, Athinoula A. Martinos Center for Biomedical Research, Massachusetts General Hospital and Harvard Medical School, Charlestown, MA, United States; b Department of Radiology, Massachusetts General Hospital and Harvard Medical School, Boston, MA, United States; c Brain and Eye Pain Imaging Lab, Department of Anesthesiology Critical Care and Pain Medicine, Boston Children’s Hospital Boston, Pain and Affective Neuroscience Center, MA, United States; d University of Miami Health System Bascom Palmer Eye Institute, Miami, FL, United States; e Surgical Services, VA Miami Healthcare System, Miami, FL, United States; f Radiology, University of Miami, Coral Gables, FL, United States; g Research Service, VA Miami Healthcare System, Miami, FL, United States; h Physical Medicine and Rehabilitation, University of Miami, Coral Gables, FL, United States; i Pediatric Pain Pathway Lab, Department of Anesthesiology Critical Care and Pain Medicine, Boston Children’s Hospital, Pain and Affective Neuroscience Center, Boston, MA, United States; j Department of Anesthesiology Critical Care and Pain Medicine, Boston Children’s Hospital, Boston, MA, United States; k Faculty Dentistry, and Department of Physiology, Temerty Faculty of Medicine, and Centre for the Study of Pain, University of Toronto, Toronto, Ontario, Canada; l Department of Ophthalmology, Boston Children’s Hospital, Boston, MA, United States

**Keywords:** Trigeminal ganglion, Ocular pain, White matter integrity, Diffusion MRI

## Abstract

Noxious stimuli to the ocular surface are encoded by sensory axons of trigeminal ganglion (TG) neurons and conveyed through the ophthalmic branch of the trigeminal nerve (CN V1). We hypothesized that chronic ocular surface pain (COSP) may be associated with microstructural alterations of the trigeminal nerve structures. Our objective was to demonstrate the feasibility of using diffusion tractography to identify and analyze diffusion properties to assess TG microstructure in individuals with and without COSP.

Forty COSP patients (27 males and 13 females; mean age: 56.2 ± 11.9 yrs; range: 34–77 yrs) and 17 controls without pain (15 males and 2 females; mean age: 55.4 ± 8.9 yrs, range: 37–66 yrs) were included in the study. Using 3T diffusion MRI (dMRI), we performed tractography to reconstruct TG and CN V1 with a generalized q-sampling imaging (GQI) algorithm. dMRI-based indices such as normalized quantitative anisotropy (NQA), fractional anisotropy (FA), mean diffusivity (MD), axial diffusivity (AD), and radial diffusivity (RD) were recorded for the right and left TG.

TG (*n* = 45) were successfully reconstructed. Fourteen participants (11 COSP, 3 controls) were excluded because TG could not be clearly visualized. A significant decrease in NQA (*p* = 0.03), MD (*p* = 0.04) and RD (*p* = 0.04) were found in the left TG, but not in the right TG (*p* = 0.40; *p* = 0.58; *p* = 0.64 respectively), in the COSP group as compared to controls. Decrease in these metrics are generally interpreted as indicators of axonal loss and fiber integrity, suggesting the presence of fiber damage in the left TG of patients with COSP.

## 1. Introduction

Ocular pain, which refers to any pain experienced in the eye, is one of the leading reasons people visit eyecare professionals (Galor et al., 2022). In the USA, >5 million patient visits for eye-related issues occurred annually from 2008 to 2019, with eye pain being the primary reason for seeking care in 42 % of outpatient visits and 67 % of emergency department visits for eye-related conditions (Shapiro et al. 2024). Eye pain can have a variety of contributors, such as instability in the tear film, increased tear osmolarity, inflammation of the ocular surface, and abnormalities in neurosensory functions, or a combination of these factors (Reyes et al. 2024). These issues can significantly impact daily activities, mental health, and overall quality of life (Sloesen et al. 2024). When pain persists for more than three months and is felt at the level of the ocular surface, it is defined as chronic ocular surface pain (COSP) (Galor et al. 2022). The severity of pain can vary from mild discomfort to extreme, intolerable pain (Sloesen et al. 2024).

COSP can originate from nociceptive, neuropathic, nociplastic, or a combination of mechanisms (Bushnell et al. 2015; Dieckmann et al. 2017; Goyal and Hamrah 2016; Patel et al. 2022). Nociceptive pain occurs when noxious stimuli affect the ocular surface. Neuropathic pain occurs due to a lesion or disease within the somatosensory nerves, which may occur at the level of the corneal and/or periocular nerve fibers (peripheral) (Mehra et al. 2020), within ascending and descending CNS tracts (central), or within the autonomic nervous system (Mitsikostas et al. 2022; Patel et al. 2022). In nociplastic pain, a clear lesion or disease cannot be identified (Galor et al. 2022).

Diffusion MRI is a leading non-invasive technique for studying structural alterations within biological tissue by observing the signal attenuation caused by the movement of water molecules (Bautin et al., 2025). Examining diffusion properties can provide insight into the microstructure of white matter and gray matter in specific brain regions (Assaf et al. 2019). Utilizing these diffusion properties, dMRI-based tractography (diffusion tractography) can reveal 3D tissue structures in vivo (Assaf et al. 2019). Diffusion tractography has been applied to the study of various disorders, such as neurodevelopmental and neurodegenerative conditions (Ahtam et al. 2019; Sullivan and Pfefferbaum 2010; Tae et al. 2018; Zhao et al. 2022), neoplasms (Celtikci et al. 2018; Ortug et al. 2022; Price et al. 2003; Yen et al. 2009), brain surgery planning and follow-up (Essayed et al. 2017; Nimsky et al. 2016; Panesar et al. 2019), psychiatric disorders (Kubicki et al. 2007; Podwalski et al. 2021; White et al. 2008), and acquired brain injury (Hulkower et al. 2013; Jang and Cho 2022; Taber et al. 2002; van Rhijn et al. 2024) as well as to map brain connectomes in healthy participants (Axer and Amunts 2022; Craddock et al. 2013; Jbabdi et al. 2015; Maier-Hein et al. 2017; Shi and Toga 2017). While some pain studies have used similar techniques (Elkholy et al. 2023; Hung et al. 2017; Kumaran et al. 2022; Mei et al. 2023; Rahimi et al. 2022; Sen et al. 2019; Shih-Ping Hung et al. 2019; Tohyama et al. 2018; Wang et al. 2023; Zhang and Furst 2022), dMRI-based methods in the field of chronic pain have not fully reached their potential (Bautin et al., 2025).

The ocular surface consists of the cornea and conjunctiva, with innervation supplied by the trigeminal nerve (CN V). Noxious stimuli are transduced by the corneal nerves and conducted through the CN V ophthalmic branch and trigeminal ganglion to the trigeminal nucleus in the brainstem. We hypothesized that COSP may be associated with alterations in the first branch of the trigeminal nerve (CN V1, ophthalmic) and trigeminal ganglion (TG), given the evidence that some individuals with COSP have neuropathic pain driven by peripheral nerve abnormalities (Aggarwal et al. 2015; Dieckmann et al. 2017; Hamrah et al. 2017; Patel et al. 2022; Theophanous et al. 2015). Prior dMRI studies have reconstructed CN V-related tracts and/or analyzed diffusion parameters in trigeminal neuralgia (TN) (Elkholy et al. 2023; Hung et al. 2017; Kumaran et al. 2022; Sen et al. 2019; Shih-Ping Hung et al. 2019; Tohyama et al. 2018; Wang et al. 2023), migraine (Rahimi et al. 2022; Mungoven et al., 2020), new daily persistent headache (Mei et al. 2023) and a variety of pain-related conditions (Zhang and Furst 2022). Some studies also aimed to anatomically reconstruct more accurate CN V and TG tracts in healthy participants (Jacquesson et al. 2020; Xie et al. 2020; Yoshino et al. 2016; Zhang et al. 2020). However, to the best of our knowledge, no studies have assessed the macro- and microstructural changes of CN V structures in COSP. Our objective was to demonstrate the feasibility of using diffusion tractography to identify TG microstructure and analyze diffusion properties in individuals with and without COSP.

## 2. Patients and methods

### 2.1. Study participants

The study was approved by the Miami Veterans Affairs (VA) and the University of Miami Institution Review Boards (IRB approvals #3011.08 and 20,190,340, respectively). The study was conducted in accordance with the principles of the Declaration of Helsinki and complied with the requirements of the United States Health Insurance Portability and Accountability Act. Written informed consent was obtained from all participants prior to any study activities.

Our study consisted of 40 participants (27 M, 13F, age 56.2 ± 11.9 yrs) who were seen at the Miami Veterans Affairs Medical Center Eye Clinic with an ocular pain complaint (symptoms present ≥3 months, average pain rating over 1-week recall ≥1 on a 0–10 numerical rating scale [NRS]), dry eye symptoms (5 Item Dry Eye Questionnaire-5 [DEQ-5] score > 6), and 17 controls (15 M, 2F, age 55.4 ± 8.9 yrs) seen within the same eye clinic for a non-pain related complaint (refractive error, etc., NRS=0). 34 of the COSP cohort also had photophobia (i.e., sensitivity to light, defined by the Ocular Surface Disease Index [OSDI], question #1 ≥ 1 out of 4 and/or Neuropathic Pain Symptom Inventory modified for the Eye (NPSI-Eye), question #9 ≥ 1 out of 10). OSDI-Q1 asks, “Have you experienced eyes that are sensitive to light during the last week?’ and NPSI-Eye-Q9 asks ‘Is your pain provoked or increased by light during the past 24 h?’. As our clinical COSP group was highly heterogenous, demographics, dry eye surveys, past ocular and medical history were collected for each participant. Participants were excluded from the study if they had ocular diseases that could confound photophobia (e.g., glaucoma, use of glaucoma medication, uveitis, iris transillumination defects, retinal degeneration, anatomical abnormalities of the cornea, conjunctiva, or eyelids, etc.), as the pathophysiology of ocular pain and photophobia in these individuals is likely different from that in the COSP group we aimed to study.

### 2.2. Measures

Participants were administered questionnaires to collect demographic and supporting health information. Individuals filled out standardized questionnaires regarding ocular symptoms, including the DEQ-5 (range 0–22) (Chalmers et al. 2010), OSDI (range 0–100) (Schiffman et al. 2000), NRS for average ocular pain intensity during the past week (range 0–10), and NPSI-Eye (range 0–100) (Farhangi et al. 2019). Individuals also completed standardized questionnaires regarding depression symptoms (patient health questionnaire-9 [PHQ-9], range 0–27). Out of all participants, three participants did not fill out the entire self-reported clinical characteristics form.

In order to evaluate the ocular surface, the participants underwent measurement of tear breakup time (TBUT) (measured in seconds, with lower values indicating tear instability, <5 is associated with severe DED, maximum 15 s), fluorescein corneal staining (graded to the National Eye Institute (NEI) scale (Lemp 1995) -higher values indicate higher irregularity in the epithelium, maximum 15), and tear production using anesthetized Schirmer strips (measured in millimeters of wetting at 5 min, - lower values indicate less tear production, <5 is extreme dry eye indicator; max is 35 mm). The laterality of the eye with COSP, pain duration, and specific timeline of medication use was not assessed.

### 2.3. Structural and diffusion MRI acquisition

Imaging was conducted using a 3T Siemens MAGNETOM Vida scanner (Erlangen, Germany). For anatomical scans, a sagittal three-dimensional T1-weighted scan (MPRAGE) was performed (TE/TR = 2.38/2100 ms; 192 1.00 mm-thick sagittal slices; in-plane resolution = 1.00 × 1.00 mm, matrix 256 × 256].

The diffusion images were acquired using: 64 different diffusion directions (*b* = 700 s/mm2), 6 non-directional diffusion-weighted imaging (*b* = 0 s/mm2), using a 2D echo planar imaging (EPI) diffusion sequence (ep2d_diff_mddw_20_p2) [TE=89 ms, and TR=9300 ms, matrix 128 × 128, through-plane slice thickness 2 mm].

### 2.4. Tractography of patient and healthy control groups

Several strategies have been developed for accurate tractography of the CN V (Danyluk et al. 2021; Jacquesson et al. 2020; Xie et al. 2020; Yoshino et al. 2016; Zhang et al. 2020). The literature suggests that deterministic tractography has some pitfalls for mapping complete CN V structures (Yoshino et al. 2016). The diffusion tensor imaging (DTI) model with deterministic, streamline tractography algorithms utilize the orientation data within voxels to trace the paths of white matter tracts (Jones 2010), and the resulting image is derived from the primary eigenvector in each voxel. For this reason, we used a q-ball based method Generalized Q-sampling Imaging-(GQI). Q-ball imaging is an advanced diffusion MRI technique that overcomes the limitations of DTI by resolving complex fiber orientations within a voxel, enabling more accurate mapping of brain connectivity especially in complex areas such as with crossing, branching, or kissing fibers that come into close contact with each other (Tuch 2004).

The whole brain tractography was reconstructed using DSIStudio software with a GQI method (Yeh et al. 2010) with a diffusion sampling length ratio of 1.25, and a deterministic tractography algorithm. FMRIB Software Library (FSL) eddy was used to correct for eddy current distortion. The correction and preprocessing were conducted through the integrated interface in DSI Studio (“Chen” release-2022 version). The accuracy of the b-table orientation was examined by comparing fiber orientations with those of a population-averaged template (Yeh et al. 2018). Restricted diffusion was quantified using restricted diffusion imaging (Yeh et al. 2017). Tensor metrics were calculated using diffusion weighted imaging (DWI) with a default setting that uses DWI with b-values lower than 1750 s/mm^2^.

### 2.5. Placement of regions of interest (ROI)

**Fig. 1.**
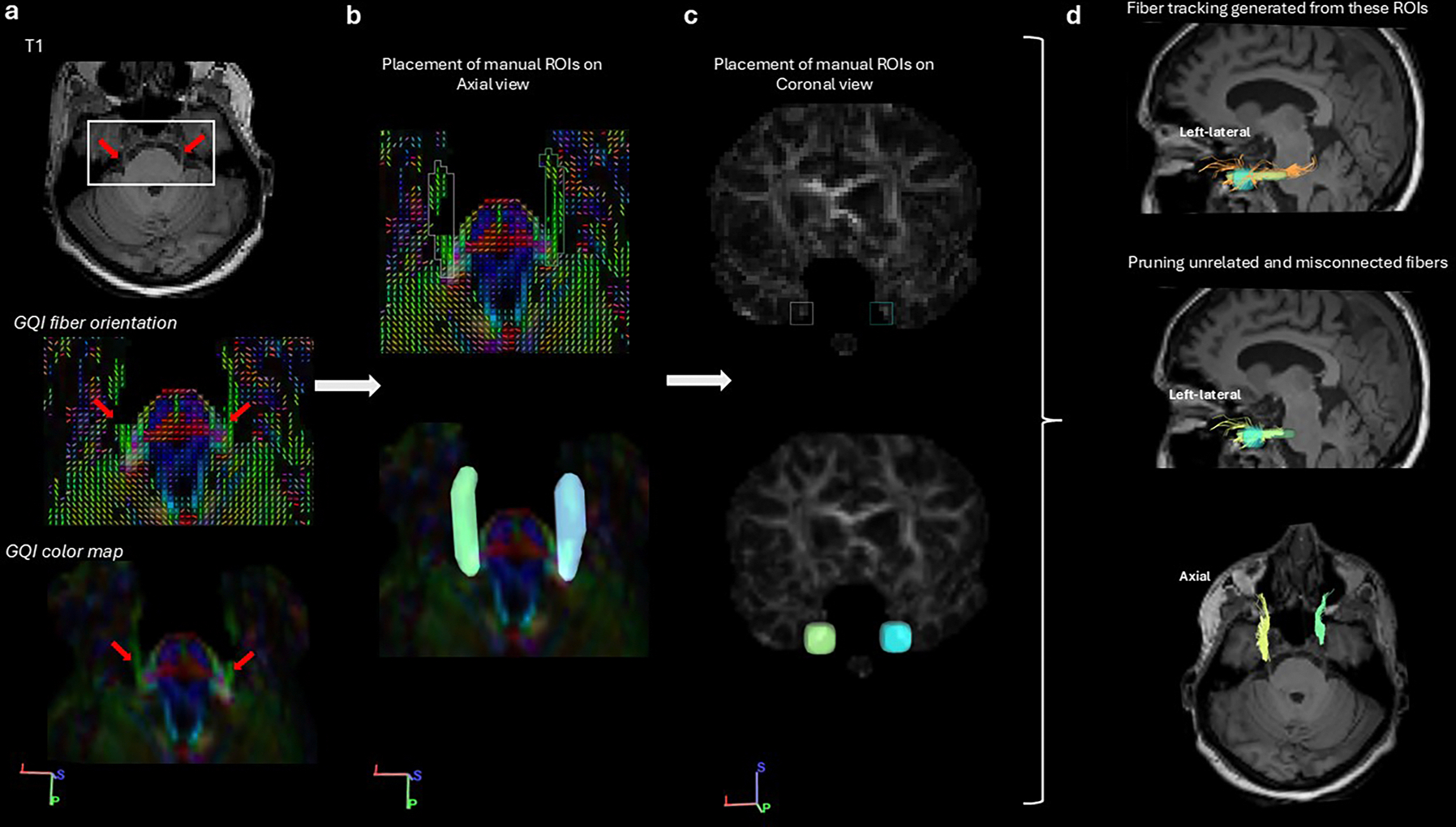
ROI placement and TG reconstruction. Column a) Identification of CN V at mid-pons level. Upper: T1 anatomical image, Middle: GQI fiber orientation on color map*, Lower: Color map, Column b) manual ROI placement in the cisternal portion of CN V, Column c) Secondary ROI placement in coronal section on Meckel’s cave on QA map, Column d) Upper: Filtering tracts using these two ROIs (left sagittal view), Middle: After virtual dissection of misconnected fibers (left sagittal view), fibers are pseudo-colored, Lower: Right (yellow) and left (green) TG on axial view. * Color maps are in standard red-green-blue (RGB) orientation, red: right-left, blue: superior-inferior, and green: anterior-posterior.

Accurate ROI placement in the brainstem is challenging because all the cranial nuclei are surrounded by other nuclei or fiber tracts. Identifying the CN V is highly dependent on the placement of the ROI, making the selection of the most effective ROIs challenging (Zhang et al. 2020). Incorrect placement of the ROI may result in false-positive tracts. Consequently, the cisternal portion of the CN V is a widely used ROI (Chen et al. 2021; Danyluk et al. 2021; Moayedi and Hodaie 2019). In order to segment the TG, regions of interest (ROI) filters were placed manually with a protocol determined with classic anatomical information and previous approaches for identifying the CN V in diffusion MRI tractography studies (Xie et al. 2020; Yoshino et al. 2016; Zhang et al. 2020). CN V is initially identified at the mid-pons level (Fig. 1a). Subsequently, the first ROI was created from the root entry zone (REZ) and cisternal portion (CP) of the CN V at the anterolateral surface of the mid-pons level where it leaves the brainstem and course through the prepontine cistern, on an axial view of a color-coded image (Fig. 1b). The second ROI was placed in the Meckel’s cave for the TG in a coronal view of quantitative anisotropy (QA) maps consistent with the existing literature (Xie et al. 2020) (Fig. 1c). While in most participants, connections between CN V and TG were not detected at the CP on the diffusion weighted images, in some participants they were identified continuously. In such cases, tractography fibers for the TG reconstruction were manually terminated at the level of porus trigeminus (PT). Tractography fibers were assessed and manually corrected for anatomical accuracy if they were connected to neighboring pathways due to streamline algorithm errors (referred to as erroneous fibers or false continuations on figures) (Fig. 1d), with identification of erroneous fibers and corrections made by tracing pathways through anatomical landmarks and referencing anatomical atlases, textbooks, and existing literature (Danyluk et al. 2021; Jacquesson et al. 2020; Paulsen et al. 2018; Xie et al. 2020; Yoshino et al. 2016; Zhang et al. 2020). The same protocol was applied bilaterally, and results were recorded separately for the right and left sides.

T1 weighted anatomical scans were registered in the DWI space using the generic rigid (all) General Registration (Elastix) (https://www.slicer.org, 3d slicer image computing platform; Slicerelastix) in 3DSlicer (Klein et al., 2010; Pieper et al., 2004; Piper et al., 2006; Shamonin et al., 2014). The registered images were utilized for anatomical reference.

**Fig. 2.**
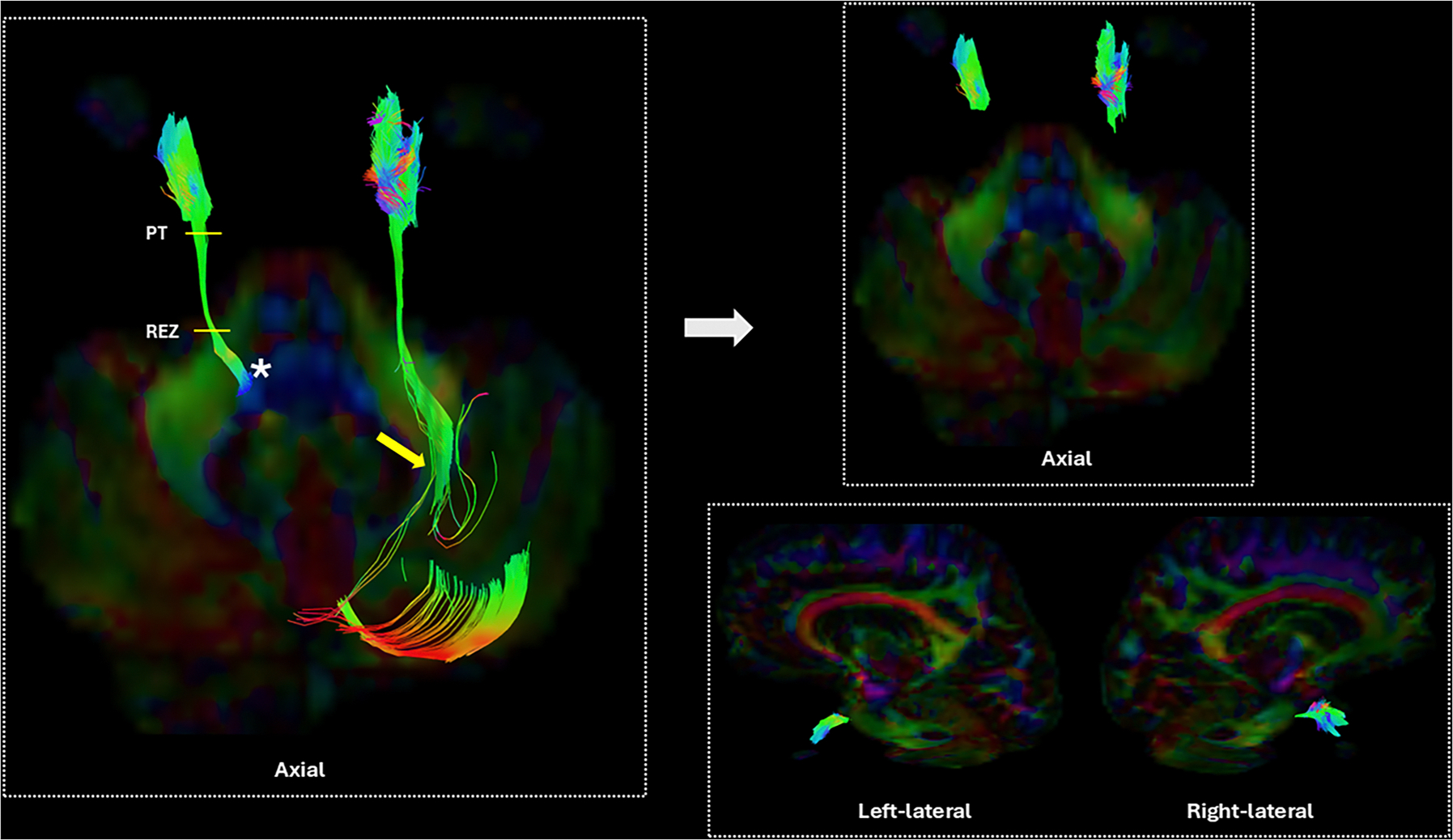
The identification of the landmarks for the REZ and PT. Left: Identification of PT and REZ for participants with the CN V continuous from the brainstem, The Asterisk shows fibers from the spinal trigeminal nucleus (SN), and the yellow arrow shows false continuation to the cerebellum. Right: Manual dissection of these fibers and reconstruction of the TG. The fibers are in standard RGB colors. (PT: Porus trigeminus, REZ: Root entry zone).

Fibers from the spinal trigeminal nucleus (SN) (Fig. 2, asterisk) were not included because of their proximity to the middle cerebellar peduncle that caused false connectivity to appear on the images between the pontocerebellar fibers and CN V through the cerebellar region in all participants (Fig. 2). The identification of the fibers through SN was also inconsistent among participants. For these reasons, we decided to limit our analysis to TG.

TGs in 45 participants were successfully reconstructed for the initial step of the study. 12 participants (9 OP, 3 controls) were excluded due to the inability to visualize TG (*n* = 11) and the algorithm’s inability to complete tractography reconstruction (*n* = 1). (In further analysis to identify CN V1, we had to exclude 2 additional participants as explained in more detail below).

The following diffusion MRI-based indices were quantified: fractional anisotropy (FA), mean diffusivity (MD), axial diffusivity (AD), and radial diffusivity (RD), as well as normalized quantitative anisotropy (NQA) which is a scaled version of quantitative anisotropy (QA) calculated from the peak orientations on a spin distribution function (SDF) (Yeh et al. 2010) [NQA = QA/ [max QA value (Bergamino et al. 2020)]. NQA minimizes inter-subject differences in spin-density and operates under the assumption that all participants have identical white matter compactness (Hong et al. 2023; Yeh et al. 2019), which can be considered an improved measure of FA.

**Fig. 3.**
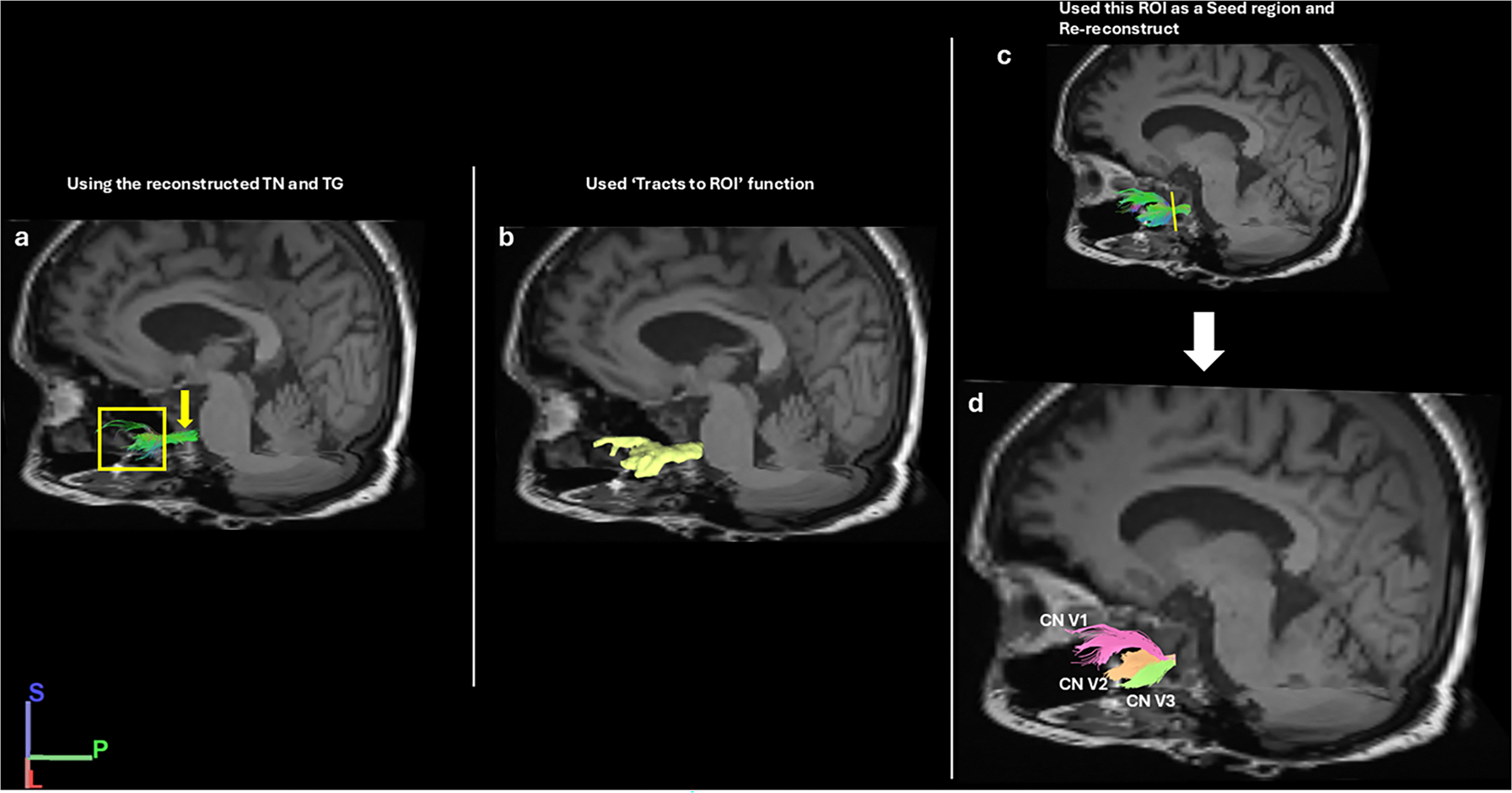
Processes for refining the branches of the TG. a) The ROI filtered TG (yellow square) and CN V (yellow arrow). b) The ROI obtained by using [Tract to ROI] function. c) Refinement of TG branches using this ROI as a seed region. Artifact fibers were eliminated; yellow line indicates the virtual dissection location to crop CN V. The fibers are in standard RGB colors. d) The CN V1 (fuchsia), CN V2 (orange), CN V3 (green) indicated separately.

In the first step, which involved “ROI filtering”, DSIStudio software used the whole brain region as the seed region (DSI-Studio) (https://dsi-studio.labsolver.org/doc/gui_t3_roi_tracking.html). After reconstructing the trigeminal nerve structures (TG and CN V) using the ROI filtering method (Fig. 3a), we then assigned the tracts from the ROI used as the seed region to refine the tracking results (“seed-based”). In the “seed-based” analysis to refine the results of the bilateral TGs and branches, we used [Tracts] and [Tract to ROI] functions of DSI studio to place seed regions (Fig. 3b). The refined results of the seeded regions were also manually corrected for erroneous streamline connections (Fig. 3c). After reconstruction of the TG, all three branches were identified (V1, V2, V3) and the results were used for qualitative analysis of the pathways (Fig. 3d). Additional steps are shown in Fig. 3.

### 2.6. Statistical analysis

SPSS 30 (I.B.M., Chicago) software was used for statistical analysis. The results are presented as mean values ± SD ($\overset{‾}{x}\;\pm \;SD$). The Shapiro-Wilks test was used to determine whether the parameters showed normal distribution or not. *p* > 0.05 was interpreted as showing normal distribution. T-tests were applied to the parameters that showed normal distribution, and Mann-Whitney U tests were applied to the parameters that did not show normal distribution. Categorical variables in Table 1 demographics of the cohort, were expressed as percentages and compared between groups using Fisher’s exact test for independent binary proportions. Adjusted P values were obtained using median regression to adjust for age and sex for quantitative features of diffusion metrics. All p-values reported are two-tailed, and *p* < 0.05 was considered statistically significant. False discovery rate control (FDR) was used for adjusting the statistical results for multiple group comparisons at a q-value (FDR adjusted/corrected P value) of 0.05 (Benjamini and Hochberg 1995; Genovese et al. 2002) using an online calculation tool (https://tools.carbocation.com/FDR ).

Pearson’s r values and corresponding P-values were computed to assess the correlation between the NRS for average ocular pain intensity over the past week (range 0–10) and NQA values, to evaluate potential differences within groups. A P-value of <0.05 was taken as significant.

To evaluate the potential of diffusion metrics to differentiate between chronic ocular surface pain (COSP) patients and no-pain controls, we conducted receiver operating characteristic (ROC) analyses. Separate ROC curves were generated for the left and right trigeminal ganglia (TG-L and TG-R). The diffusion metrics evaluated included NQA, FA, MD, AD, and RD.

For each ROC analysis, the COSP group was coded as the positive class (group = 1), and group coding was adjusted accordingly based on whether the diffusion metric was higher or lower in COSP to ensure accurate directionality of the AUC. The area under the ROC curve (AUC), also referred to as the c-statistic, was calculated to quantify the discriminative performance of each metric. The statistical significance of the AUC values corresponds to the Mann-Whitney U test, as the nonparametric AUC estimation is mathematically equivalent to this rank-based comparison.

## 3. Results

### 3.1. Demographics of the cohort

Forty COSP patients (27 males and 13 females) and 17 controls (15 males and 2 females) were included in the study. The demographics, self-reported clinical characteristics, and medication use of all participants are given in Table 1.

The case and patient groups were similar in terms of age, sex, race and ethnicity. Ocular symptoms were also recorded on questionnaires. All dry eye, light sensitivity, and ocular pain questionnaire scores are summarized in Table 2 and were higher in cases than controls. Some of the participants, regardless of their group, had refractive, cataract, or other ophthalmological surgeries or procedures. The surgery type and laterality information are provided in Supplementary Table 1.

### 3.2. Qualitative features

**Fig. 4.**
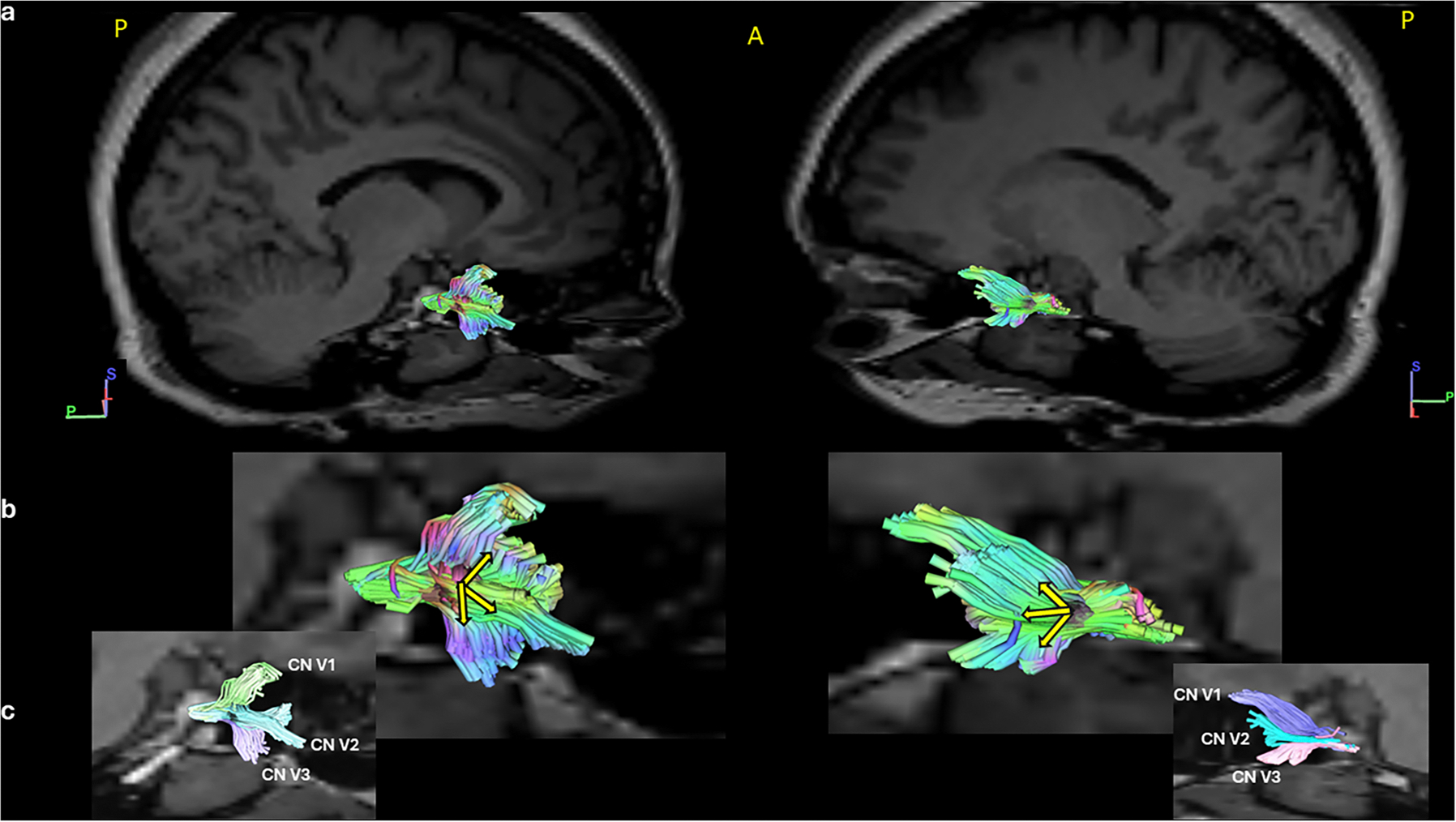
A successful reconstruction of the TG and the branches of CN V. Row a) Right and left TG overlayed on the T1 image. Row b) The right and left TG isolated-shown in standard color code. The yellow arrows show the orientation of the nerve fibers. Row c) The left and right CN V1, V2, V3 shown in different pseudo-colors. The fibers are in standard RGB colors in row a and b.

**Fig. 5.**
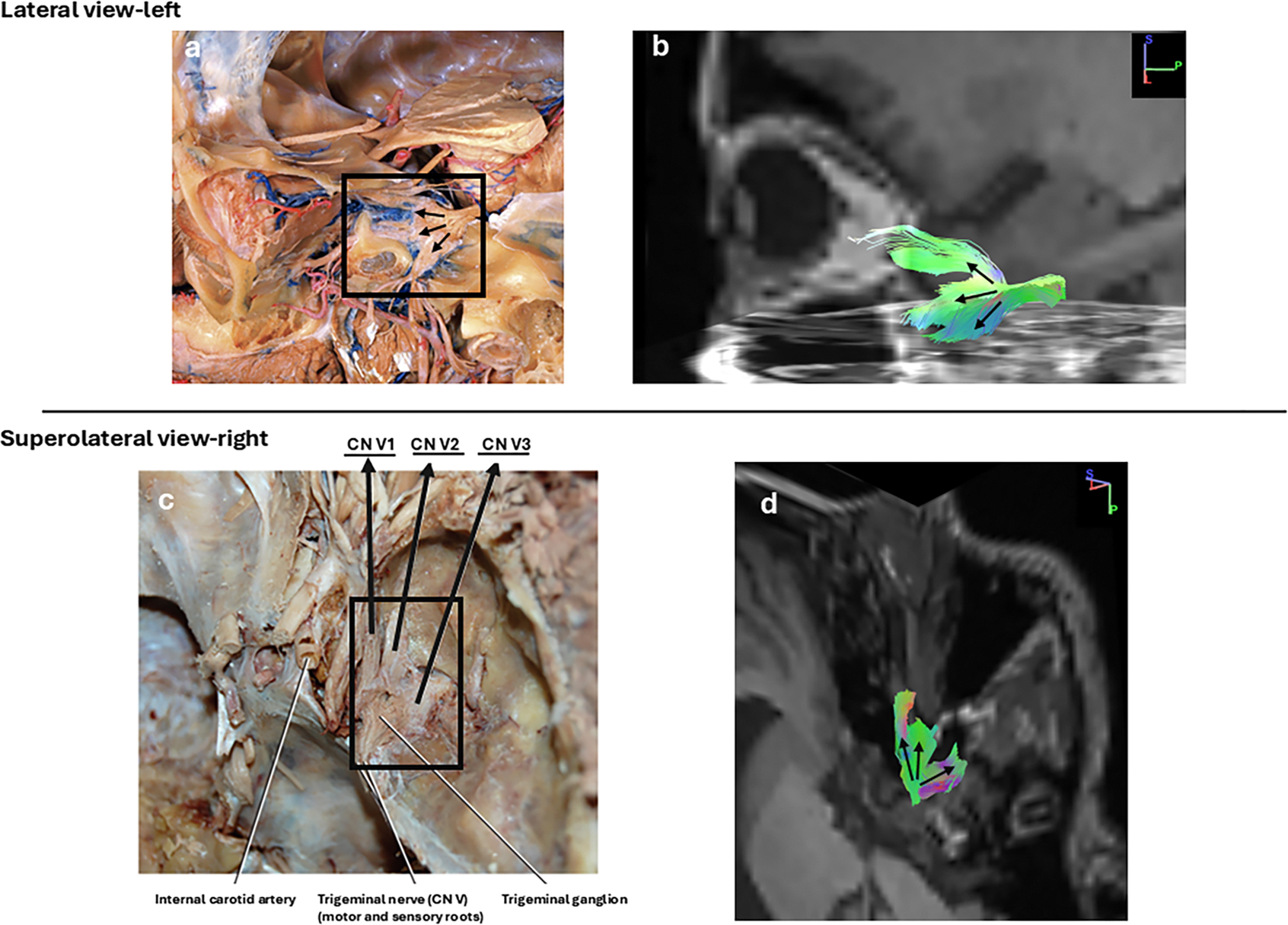
A comparison of the cadaveric and virtual dissection of the TG/CN V. Upper row: a) a cadaveric image of the left trigeminal ganglion and nerve, dissection of the left orbit from the lateral approach (Image Credit: Bassett Collection of Stereoscopic Images of Human Anatomy, Stanford Medical History Center, licensed under CC-BY-NC-SA) b) our tractography image in the same orientation. Bottom row: Right side from another angle. c) Image credit: Loukas, M., Brion B., and Tubbs RS. Gray’s Clinical Photographic Dissector of the Human Body. (2013) Used with permission Elsevier, permission conveyed through Copyright Clearance Center, Inc (Loukas et al. 2013). d) our tractography image in the same orientation.

The exit/entrance of the CN V from the anterolateral surface of the pons was visible in all 56 participants that whole brain tractography successfully created. However, TG could not be identified in 11 of these participants. False continuations into the middle cerebellar peduncle were observed in the brainstem of all participants. The spinal trigeminal nucleus/tract was also identified in some participants (*n* = 10). However, as our focus of interest was TG and the number of correct reconstructions was not consistent, we did not include that region. The successful reconstruction of TG and its branches was not influenced by whether the participants were part of the COSP or the control group. Fig. 4 shows a successful reconstruction of the TG and the branches with the seed-based method and in Fig. 5, a comparison of cadaveric and virtual dissection can be seen. No statistically significant differences in demographics or pain characteristics were detected between reconstructable (included) and non-reconstructable (excluded) participants

### 3.3. Quantitative features -Diffusion metrics

We quantified the NQA, FA, MD, AD and RD of the left and right TG for 31 COSP cases and 14 controls for the results acquired by ROI filtering. The diffusivity calculated in DSI Studio has a unit of 10^–3 mm^2/s.

We adjusted the P values for age and sex using median regression. For the group analysis between COSP cases and controls, the statistics for the TG-L and TG-R of the diffusion metrics of NQA, FA, MD, AD and RD (median (interquartile range and *p*-values) are listed in Table 3.

According to uncorrected results, diffusion parameters for TG-R were similar between cases and controls. However, individuals with COSP had significantly decreased NQA (*p* = 0.04), MD (*p* = 0.04) and RD (*p* = 0.04) compared to controls in TG-L. However, these results did not survive after the FDR correction for multiple comparisons.

**Fig. 6.**
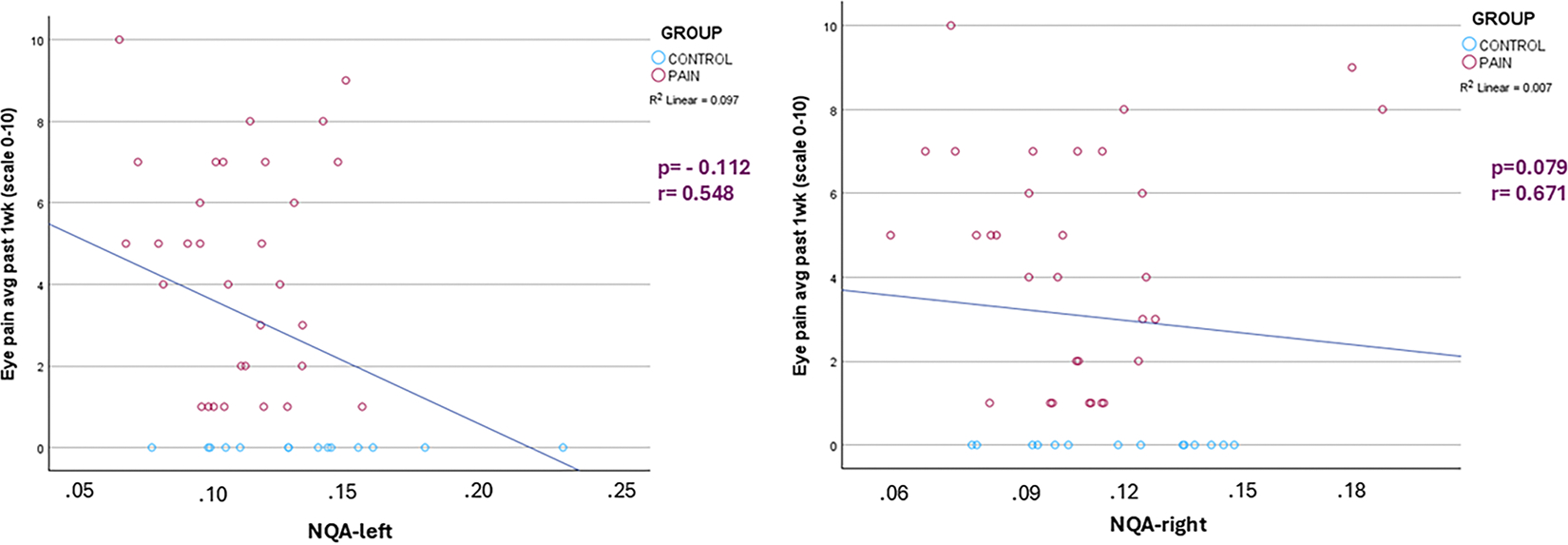
Correlation plots between average eye pain over the last week measure and NQA-left (left) and NQA-right (right) (p and r values given for COSP group. For the controls, the Pearson correlation coefficient cannot be computed as the average eye pain over the last week is constant (=0).

In the control group, the correlation between eye pain average over the last week (0–10) and both NQA-left and NQA-right could not be computed due to the lack of variability in at least one of the variables (Fig. 6). In the pain group, the correlation between eye pain average over the last week (0–10) and NQA-left was very weak and negative (*r* = −0.112, *p* = 0.548), and it was not statistically significant (Fig. 6, left). The correlation between eye pain average over the last week (0–10) and NQA-right was weak and positive (*r* = 0.079, *p* = 0.671), with no statistical significance (Fig. 6-right). These results suggest that there is no meaningful relationship between eye pain and either NQA-left or NQA-right in the pain group. Both correlations indicate very weak tendencies with no significant association.

ROC analyses were conducted separately for the left and right trigeminal ganglia (TG) using five diffusion metrics: NQA, FA, MD, AD, and RD.

For the left TG, NQA demonstrated the strongest discriminatory performance, with an AUC of 0.703 (*p* = 0.024), reflecting significantly lower values in the COSP group compared to controls (see Table 3 for group comparisons, and Table 4 for ROC summary). Similarly, MD, AD, and RD also showed significantly lower values in the COSP group and resulted with AUCs of 0.717 (*p* = 0.009), 0.687 (*p* = 0.028), and 0.721 (*p* = 0.008), respectively. FA, which did not differ significantly between groups, had an AUC of 0.417 (*p* = 0.345).

For the right TG, all metrics showed non-significant discriminatory performance. Right sided NQA had an AUC of 0.645 (*p* = 0.123). Other metrics—including FA, MD, AD, and RD—had AUCs ranging from 0.488 to 0.585, none of them reached statistical significance.

These findings suggest that while right-sided metrics had limited ability to distinguish COSP from controls, left-sided NQA, MD, AD, and RD demonstrated moderate discriminative utility. These results may reflect subtle microstructural alterations in the left trigeminal ganglion associated with chronic ocular surface pain. ROC curves for all metrics are presented in Supplementary Figure 1.

## 4. Discussion

To our knowledge, this is the first study to assess the trigeminal ganglia (TG) quantitatively and ophthalmic nerves (CN V1) qualitatively in individuals with chronic ocular surface pain (COSP), using diffusion MRI tractography. Importantly, all data were acquired using a clinical 3T scanner with standard imaging protocols, underscoring the translational feasibility of detecting trigeminal microstructural changes in routine clinical settings. In this study, we successfully reconstructed TG and CN V1. We quantified and analyzed microsctructural alterations in TG using diffusion metrics and also qualitatively assessed CN V1 for most of our participants in both cohorts. Our findings reveal significant microstructural alterations in the TG associated with the presence of COSP, with the key finding being a unilateral (left-sided) decrease in normalized quantitative anisotropy (NQA), mean diffusivity (MD) and radial diffusivity (RD) in the TG in COSP patients compared to controls. This decrease may indicate reduced directional diffusivity in the tissue (e.g., axonal loss and fiber damage) as well as reduced mean diffusivity in COSP. This aligns with prior studies suggesting that reduced NQA is a biomarker of fiber injury (Huang et al. 2023) and decreased MD and RD a sign of pathological changes affecting both the overall tissue structure and myelin integrity (Solowij et al. 2017; Song et al. 2002). Given that neuropathic pain is characterized by damage to the somatosensory nervous system, these microstructural alterations could contribute to the underlying mechanism of chronic pain in our cohort.

### 4.1. Reduced diffusion metrics and comparison with existing literature with CN V-related pain

Our findings have biological relevance to instances where the cornea and ocular surface are injured or inflamed, and peripheral axons of the ophthalmic branch of the trigeminal nerve become activated. The exposure to a persistent barrage of noxious signals may predispose the TG and CN V1 to damage, which in our study was potentially captured by the findings of a decreased NQA, MD and RD. Prior studies have examined dMRI metrics in chronic head and facial pain conditions, including temporomandibular disorders (TMD) and trigeminal neuralgia (TN). Similar to our findings, these studies also reported alterations in some of the diffusion metrics in the CN V of individuals with TMD (Moayedi et al., 2012). Specifically, TMD patients exhibited significantly lower fractional anisotropy (FA), and higher mean diffusivity (MD) and radial diffusivity (RD) in the bilateral CN V, indicating generally compromised white matter integrity from damage such as axonal loss, demyelination, or increased extracellular space (Aung et al. 2013; Beaulieu 2002). These findings were interpreted by the authors as a result of increased nociceptive firing – either from peripheral sources or from aberrant firing patterns – which could gradually impact the microstructure of the trigeminal nerve and contribute to central abnormalities along the ascending nociceptive system (Moayedi et al., 2012).

Previous studies have used dMRI also to examine TN (Chen et al. 2019; DeSouza et al. 2014; Koh et al. 2021; Leal 2019; Leal et al. 2011; Moon et al. 2018; Pikis et al. 2021; Shapey et al. 2019; Shih-Ping Hung et al. 2019; Su et al. 2024; Tohyama et al. 2018; Willsey et al. 2020; Wu et al. 2020; Zhang et al. 2018) and some of these have reported changes in specific dMRI metrics (Cauley and Filippi 2013; DeSouza et al. 2014; Elkholy et al. 2023; Fujiwara et al. 2011). TN and COSP may be related through shared dysfunction of the CN V, which can lead to altered sensory processing, nerve damage, or central sensitization, contributing to chronic pain in both conditions. Consequently, dMRI findings from TN studies can inform the interpretation of our COSP results. Consistent results across these studies included decreased FA (Elkholy et al. 2023; Herweh et al. 2007) and increased MD, AD and RD, primarily focusing on the root entry zone (REZ) on the symptomatic side in TN patients (DeSouza et al. 2014; Leal 2019; Moon et al. 2018; Willsey et al. 2020), with one exception that found no changes in diffusion measures (Wilcox et al. 2013). A review by Shapey et al. highlighted variability in scanning parameters, regions of interest (ROIs), tractography methods, and key findings (see Tables 1–2 of (Shapey et al. 2019) for studies in TN and other cranial nerves). These changes in diffusion measures indicate decreased water diffusivity in the tissue. Considering NQA is a theoretically improved measure of FA (see 2.4), NQA results appear to be consistent with these previous findings except for our observed decreases in MD and RD which do not align with prior reports. This discrepancy could potentially arise from both biological and technical factors, such as differing mechanisms between TMD, TN, and COSP, or variations in ROI locations, methodologies, and scanner parameters (Shapey et al. 2019).

### 4.2. Heterogeneity of diffusion metrics results

Diffusion MRI-based metrics can provide insights into the microstructural features of tissue. However, quantitative diffusion MRI results may vary depending on the methodology used in each research study, so caution is needed when interpreting results across studies. DTI indices are based on tensor models, which inevitably have limitations based on DTI methodology, identifying a maximum of one diffusion direction within each imaging voxel (Tuch et al. 2003). These indices can still show association with microstructures - for example, FA was linked to axonal integrity and decreases with demyelination, inflammation, edema, and axonal loss (Chang et al. 2017); similarly, alterations in AD, RD and MD are associated with changes in axonal density (Song et al. 2003; Song et al. 2002), demyelination (Budde et al. 2009; Song et al. 2003; Song et al. 2002), and inflammation/edema (Beaulieu 2002), respectively.

However, because these indices assume that the direction of water diffusivity detected within each voxel is limited to one direction, it is difficult to accurately detect complex and varied factors. Recently, the generalized q-sampling imaging (GQI) method has emerged as an improved version of DTI techniques with a model-free approach that calculates the spin distribution function (SDF) from diffusion MR signals. GQI can differentiate between restricted and unrestricted diffusion through the use of both anisotropic and isotropic measures (Yeh et al. 2010), thus incorporating diffusivities in multiple directions. The QA index from the GQI method is considered more robust due to its reduced sensitivity to partial volume effects from crossing fibers and free water (Yeh et al., 2013). Additionally, QA is linked to axonal density and is less affected by edema (Yeh et al. 2013). Compared to FA, QA’s primary advantage lies in its ability to distinguish not just a single fiber within a voxel but the entire fiber group, leading to more accurately reconstructed images (Peled et al. 2006; Yeh et al. 2010). This capability may explain why we observed a statistically significant decrease in NQA specifically but not in FA, given the complex tissue structure of the TG and its branches.

### 4.3. Laterality of pain

Our results indicated a significant decrease in normalized QA (NQA) only in the left TG in the COSP group. Unfortunately, the laterality of pain symptoms were not documented. Nevertheless, the lack of bilateral changes is surprising given that unilateral trigeminal nerve injuries are typically accompanied by bilateral structural abnormalities in TG and trigeminal brainstem regions (DeSouza et al. 2014; Desouza et al. 2013). Lateralization effects have been observed in TN, where pain predominantly affects the right side of the face (Bangash 2011; Obermann 2012; Toda 2009). In addition, multiple studies have reported that unilateral ocular lesions can also affect the contralateral eye (Gong et al. 2024). There are two leading hypotheses to explain this (Gong et al. 2024). One theory suggests that unilateral nerve damage triggers central nervous system sensitization, leading to contralateral nerve involvement through central regulation (Hamrah et al. 2010), another proposes that the trigeminal pathway crosses the midline to reach the contralateral main nucleus (Gong et al. 2024; Oaklander et al. 1998).

A related consideration is whether left V1 or V2 afferents could cross the midline and contribute to right eye pain, or vice versa, similar to how anterior teeth can receive bilateral CN V2 or CN V3 innervation. Animal studies have demonstrated a similar mechanism, showing that inferior corneal innervation originates from the maxillary nerve (Müller et al. 2003; Ruskell 1974). In humans, a comparable morphological variation, termed ‘dento-corneal aberrant regeneration,’ has been described in case reports as a minor contributor to inferior corneal innervation (Bery and Fraser 2017; Vonderahe 1928). The frequent bilateral effects following unilateral nerve damage or inflammation in orofacial pain models reinforce the need to investigate potential cross-midline trigeminal innervation in future studies.

Although the spinal trigeminal nucleus (SN) was excluded from our tractography analysis due to technical limitations related to fiber tracking and its proximity to the middle cerebellar peduncle, it is important to note that the SN plays a key role in processing nociceptive inputs from surface tissues, including the cornea. Nash et al. reported that the trigeminothalamic tract can be activated bilaterally in response to orofacial pain following unilateral (right-sided) injections of hypertonic saline into the masseter muscle (Nash et al. 2010). In addition, an earlier study from the same group showed that during cutaneous pain, signal intensity increased throughout the rostrocaudal extent of the SN — involving the oralis (SpVo), interpolaris (SpVi), and caudalis (SpVc) subdivisions — whereas muscle pain activated only discrete regions of SpVo and SpVc, without involving SpVi (Nash et al. 2009). The cornea contains both polymodal nociceptors, which actively generate action potentials at their sensory nerve endings, and cold-sensitive receptors, whose signals are passively conducted from a more proximal point along the axon (Brock et al. 2001). This dual mechanism of nociceptive signal transmission from different types of corneal receptors shows that the spinal trigeminal nucleus may play an important role in corneal sensory processing and should be considered when studying nociceptive pathways. While the spinal trigeminal nucleus is clearly an important component of the nociceptive pathway involved in corneal pain, the diffusion approach implemented was not able to cleanly discern fibers en route to this nucleus vs. nearby fibers within the middle cerebellar peduncle.

Our COSP group exhibited considerable heterogeneity in self-reported pain, duration, and intensity. To examine the relationship between self-reported pain levels and NQA values, we conducted a correlation analysis. However, no significant correlation was observed between the average pain reported over the last week and NQA levels on either the left or right side. The diversity within the pain groups may also explain the heterogeneous effects observed in the overall statistical analysis of the right and left trigeminal ganglia (TG). It is widely recognized that the body activates compensatory mechanisms in response to threats, helping to mitigate effects and restore balance (Kobrzycka et al. 2019). The unilateral results may suggest a compensatory mechanism that has evolved as an adaptation to chronic pain in the COSP group. While no lateralization of function is expected at the level of the trigeminal nerve, future studies on COSP should specifically address the observed left, sided changes and capture severity of pain complaints in each eye separately.

### 4.4. Interpretation of results and assessment with the molecular background of the COSP

Injuries to the corneal surface have been associated with molecular changes in the peripheral and central mechanisms of the trigeminal complex, primarily studied in animal models (Ferrari et al. 2014; Huang et al. 2021; Katagiri et al. 2023; Meng and Bereiter 1996; Tashiro et al. 2010). Corneal nerve injury, which can result from various insults, has been explored using models such as chronic exposure to benzalkonium chloride (BAC) (Liang et al. 2012; Lin et al. 2011; Sarkar et al. 2012), where a dose-dependent neurotoxic effect of BAC was linked to a significant decrease in nerve fibers in primary mouse trigeminal ganglion cultures (Launay et al. 2016).

Changes in the density and morphology of subbasal corneal nerves can be observed in different pathological conditions (Müller et al. 2003). Several in vivo confocal microscopy (IVCM) studies have examined corneal nerve anatomy in various dry eye patients, including those with Sjögren and non-Sjögren diseases. Many of these studies reported decreased nerve density in the subbasal corneal nerve plexus compared to control participants (Cruzat et al. 2017; Guerrero-Moreno et al. 2021). Additionally, there were differences in nerve morphology in individuals with COSP related to DED, with many reporting increased tortuosity compared to controls (Cruzat et al. 2017). A previous study suggested that possible tortuosity of the nerve may also create partial volume effects on dMRI (Gerlach et al. 2017), which also indirectly affects the diffusion metrics (Vos et al. 2011). Thus, our decreased NQA results could also be indirectly related to abnormal nerve morphology.

The noted morphological changes at the level of the cornea in various populations of individuals with dry eye, who can have COSP, highlight that alterations may also occur higher up in the CN V and may be captured by dMRI technology. The reduction in NQA, MD and RD in COSP patients suggests disruptions in microstructural organization within the central trigeminal complex, linking peripheral nerve injury to central neuroanatomical changes detectable by advanced imaging techniques like dMRI.

## 5. Limitations and future work

Our study has limitations that need to be considered. The study population was heterogenous with respect to demographics, comorbidities, and ocular features. Due to the low number of participants, we are unable to assess the contributions of these factors to our findings. First, while chronic pain conditions are known to exhibit significant sex differences, with many being more prevalent in females, we were unable to analyze such differences due to the limited sample size. Future studies should aim to recruit sufficiently large and balanced samples to explore these effects.

In addition, our initial questionnaires did not differentiate between bilateral and unilateral eye pain, and not all patients had a recorded duration of their pain. We are addressing these limitations in ongoing studies, as the heterogeneous nature of COSP necessitates subgrouping individuals according to their suspected underlying pathophysiology.

The CN V1 was only assessed qualitatively since from a technological perspective, reconstructing CN V presents challenges. This region is susceptible to imaging artifacts and noise due to the complex structure of the skull base, which contains nerves, bone, air, soft tissue, and cerebrospinal fluid (Xie et al. 2020). For this reason, some scans were excluded from analysis due to image artifacts or low reconstruction quality. We are hopeful that advancements in imaging techniques will help mitigate these issues, leading to fewer exclusions in future analyses.

Additionally, limitations inherent to the interpretation of diffusion MRI findings should be acknowledged. While altered diffusion metrics in the trigeminal ganglion are suggestive of microstructural changes, diffusion metrics are non-specific and cannot directly differentiate between axonal loss, demyelination, or inflammation. Moreover, our study is cross-sectional and thus cannot determine whether microstructural changes preceded the development of chronic ocular surface pain or arose as a consequence of chronic pain states. Finally, without histopathological validation, which is not possible in vivo, it remains challenging to directly link imaging findings to specific biological mechanisms.

Finally, the relatively small sample size limited our ability to apply more advanced statistical models without risking overfitting or reduced statistical power. While we implemented ROC analyses to explore group-level differentiation, the scope of our analysis remained univariate. Future studies with larger cohorts will be needed to support more sophisticated multivariate approaches. Additionally, combining multiple diffusion metrics through multivariable modeling or machine learning techniques may further enhance discriminative performance and should be considered in future work.

Despite these limitations, our study serves as an initial step toward assessing the trigeminal nerve complex in COSP. We believe that brainstem-focused diffusion-weighted imaging in future studies holds significant potential for uncovering underlying microstructural changes in these structures.

## 6. Conclusions

This is the first study to investigate white matter alterations in the TG in cases of COSP, compared to a control group, using diffusion tractography. We identified microstructural changes indicative of potential fiber damage in COSP, highlighting the potential of this technology for exploration of COSP subtypes in future studies.

## Supplementary Material

MMC1

MMC2

Supplementary materials

Supplementary material associated with this article can be found, in the online version, at doi:10.1016/j.neuroimage.2025.121309.

## Figures and Tables

**Table 1:** Demographics, self-reported clinical characteristics, and medication use of participants.

	Case patients (*n* = 40)	Controls (*n* = 17)	P value

**Demographics**			
Age (mean ± SD; years)	56.2 ± 11.9	55.4 ± 8.9	0.80
Sex, male % (n)	67.5 % (27)	88.2 % (15)	0.10
Race, white % (n)	67.5 % (27)	70.5 % (12)	0.82
Ethnicity, Hispanic % (n)	50 % (20)	47 % (8)	0.84
**Self-reported clinical characteristics**			
Diabetes mellitus, % (n)	12.5 % (5)	17.6 % (3)	0.61
PTSD, % (n)	40 % (16)	29.4 % (5)	0.45
Depression, % (n)	57.5 % (23)	58.8 % (10)	0.92
Arthritis, % (n)	35 % (14)	17.6 % (3)	0.19
Sleep apnea/CPAP % (n)	55 % (22)	47 % (8)	0.83
Migraine, % (n)	30 % (12)	11.7 % (2)	0.14
Traumatic brain injury, % (n)	12.5 % (5)	5.8 % (1)	0.46
Past or current smoker, % (n)	55 % (22)	70.5 % (12)	0.38
**Self-reported medication use**			
Anti-depressant % (n)	45 % (18)	29.4 % (5)	0.35
Anxiolytic % (n)	37.5 (15)	29.4 % (5)	0.66
Analgesic % (n)	60 % (24)	17.6 % (3)	**0.002**
gabapentin (Neurontin) % (n)	22.5 % (9)	11.7 % (2)	0.40
Pregabalin % (n)	5 % (2)	0 % (0)	0.36
**History of Surgery related to ocular area**			
Refractive surgery (LASIK OU,	12.8 % (5)	5.9 % (1)	0.66
PRK OU)			
Cataract surgery	12.8 % (5)	5.9 % (1)	0.66
Other ophthalmological surgery/	23.0 % (9)	5.9 % (1)	0.25
procedure			
Any ophthalmic surgery	30.7 % (12)	5.9 % (1)	0.08

* PTSD = post-traumatic stress disorder.

**Table 2:** Ocular symptoms and signs of cases and controls.

	Case patients (*n* = 40)	Controls (*n* = 17)	P Value

**DE, light sensitivity, and ocular pain symptoms assessed via questionnaires, mean ± SD (n)**			
DEQ5 (range 0–22)	13.6 ± 4.3	4.2 ± 3	<0.0001
Light sensitivity (OSDI-Q1) (range 0–4)	2.3 ± 1.5	0.9 ± 1.3	0.002
OSDI total (range 0–100)	47.9 ± 25.4	15.0 ± 20.4	<0.0001
Light sensitivity (NPSI-Eye-Q9) (range 0–10)	6.2 ± 5.3	0 ± 0	<0.0001
NSPI-Eye total (range 0–100)	29.6 ± 21.8	0.8 ± 1.3	<0.0001
Average pain rating 1 week recall (range 0–10)	4.7 ± 2.7	0 ± 0	<0.0001
**Tear parameters**			
TBUT R (mean ± SD; seconds) (n)	7.5 ± 3.8	11.1 ± 3.4	<0.01
TBUT L (mean ± SD; seconds) (n)	8.6 ± 4.1	10.7 ± 4.4	0.09
Staining R (mean ± SD; range 0–3) (n)	2.3 ± 2.9	2.3 ± 4.2	0.57
Staining L (mean ± SD; range 0–3) (n)	2.7 ± 3.1	2.1 ± 3.0	0.39
Schirmer’s R (mean ± SD; mm) (n)	13.2 ± 9.4	18.5 ± 10.2	0.1
Schirmer’s L (mean ± SD; mm) (n)	13.0 ± 9. 4	18.3 ± 9.4	0.5

SD= standard deviation, *n*= number of participants, DE: Dry eye, DEQ5 = 5 Item Dry Eye Questionnaire; NPSI-Eye = Neuropathic Pain Symptom Inventory–modified for the Eye; OSDI = Ocular Surface Disease Index; OSDI-Q1 = OSDI #1 ‘Have you experienced eyes that are sensitive to light during the last week?’; NPSI-Eye-Q9 = NPSI-Eye Question #9 ‘ Is your pain provoked or increased by light during the past 24 h?’; TBUT = tear breakup time; *R* = right; *L* = left.

* Two cases and one control did not complete the Tear parameters assessments.

**Table 3:** Statistical results for the group comparisons of TG-L and TG-R between COSP and control groups.

Analysis of Eye Pain and Trigeminal Ganglia: TG-L and TG-R					
Variable	COSP Group (*n* = 31)	Control Group (*n* = 14)	Unadjusted P value	Adjusted P value	Benjamini-Hochberg Adjusted P value

TG-L					
NQA	0.11 (0.09, 0.13)	0.13 (0.1, 0.15)	**0.03** *	**0.04** *	0.07
FA	0.19 (0.15, 0.23)	0.19 (0.15, 0.2)	0.38	0.25	0.25
MD	1.76 (1.4, 2.03)	2.06 (1.82,2.4)	**0.02** *	**0.04** *	0.07
AD	2.11 (1.71, 2.44)	2.46 (2.18, 2.93)	**0.05** *	0.19	0.24
RD	1.59 (1.22, 1.83)	1.86 (1.62, 2.15)	**0.02** *	**0.04** *	0.07
TG-R					
NQA	0.1 (0.08, 0.12)	0.12 (0.09, 0.14)	0.12	0.40	0.67
FA	0.17 (0.15, 0.18)	0.16 (0.14, 0.17)	0.36	0.60	0.67
MD	1.72 (1.42, 1.96)	1.67 (1.5, 1.88)	0.92	0.58	0.67
AD	2.05 (1.66, 2.33)	1.94 (1.74, 2.21)	0.71	0.67	0.67
RD	1.57 (1.26, 1.77)	1.51 (1.37, 1.71)	0.90	0.64	0.67

Data are presented as median (interquartile range).

Unadjusted P values were calculated using the Mann-Whitney U test.

Adjusted P values were obtained using median regression to adjust for age and sex.

* Statistically significant, *p* < 0.05.

NQA = normalized quantitative anisotropy; FA = fractional anisotropy; MD = mean diffusivity; AD = axial diffusivity; RD = radial diffusivity.

**Table 4:** ROC analysis results for diffusion metrics of the left and right TG in patients with COSP and controls. AUC values reflect the ability of each metric to discriminate between groups, with COSP coded as the positive class. Direction in COSP indicates whether the median value was higher (↑), lower (↓), or not different (↔) compared to controls.

Variable	AUC	95 % CI	p-value	Direction in COSP

**NQA-L**	0.703	0.527–0.878	**0.024**	↓
**FA-L**	0.417	0.245–0.589	0.345	↔
**MD-L**	0.717	0.554–0.879	**0.009**	↓
**AD-L**	0.687	0.520–0.853	**0.028**	↓
**RD-L**	0.721	0.559–0.884	**0.008**	↓
**NQA-R**	0.645	0.461–0.830	0.123	↓
**FA-R**	0.585	0.416–0.754	0.323	↑
**MD-R**	0.509	0.337–0.682	0.917	↑
**AD-R**	0.535	0.361–0.708	0.696	↑
**RD-R**	0.488	0.315–0.662	0.897	↑

## Data Availability

The data that has been used is confidential.
