# Supplementary figures and images for: Microstructural alterations of the trigeminal ganglion in chronic ocular surface pain patients: A diffusion MRI study
Source: Neuroimage. Author manuscript; Available in PMC 2025 Aug 15. (PMC12356230; doi:10.1016/j.neuroimage.2025.121309)

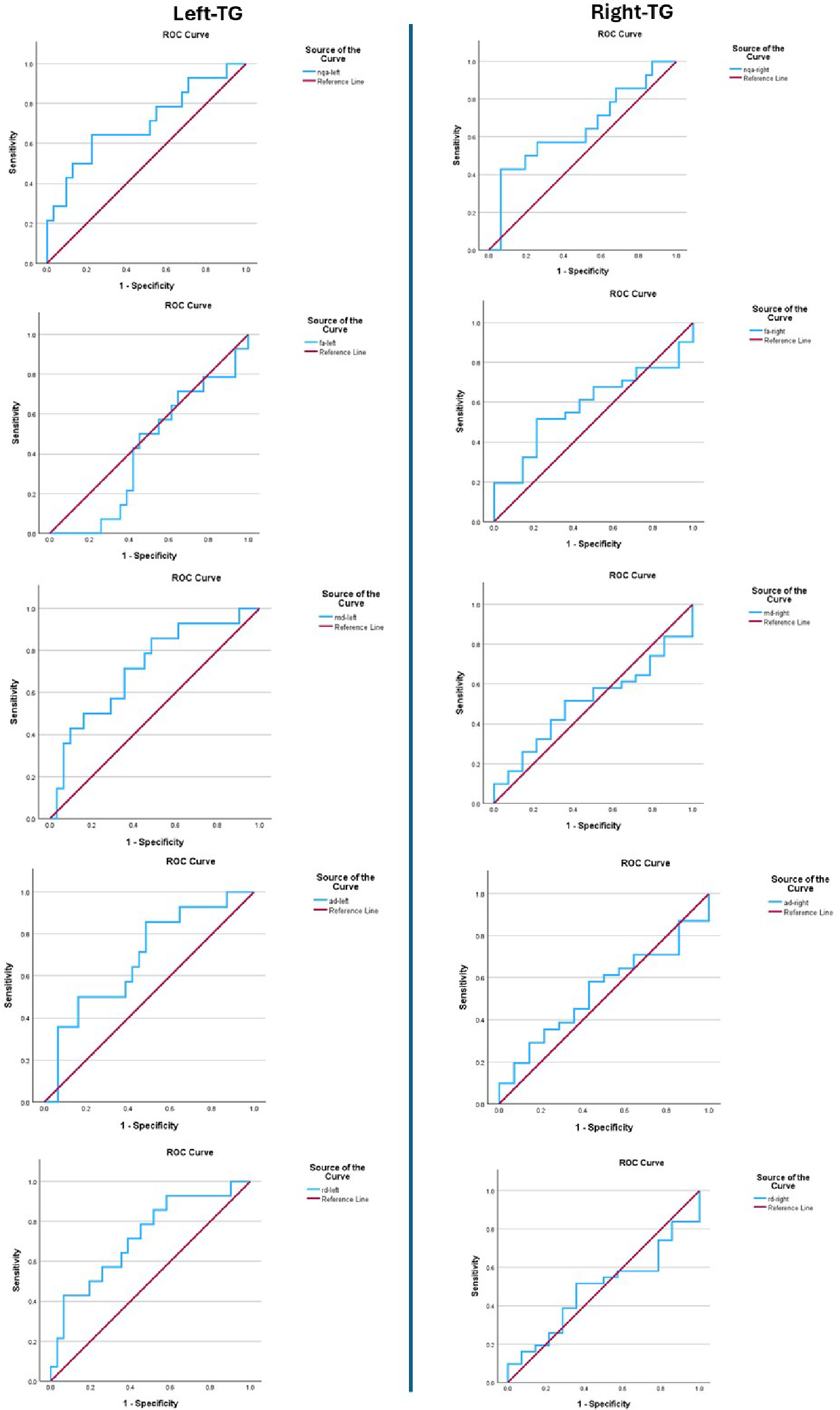

Supplement: MMC2 [file NIHMS2100748-supplement-MMC2.jpg]
